# Redetermined crystal structure of α-dl-me­thio­nine at 340 K

**DOI:** 10.1107/S1600536814022211

**Published:** 2014-10-15

**Authors:** Carl Henrik Görbitz, Lianglin Qi, Ngan Thi Kim Mai, Håvard Kristiansen

**Affiliations:** a Department of Chemistry, University of Oslo, PO Box 1033 Blindern, N-0315 Oslo, Norway

**Keywords:** crystal structure, hydrogen bonding, phase transition, disorder, zwitterions

## Abstract

Two forms, α and β, are known for the racemic amino acid dl-me­thio­nine, C_5_H_11_NO_2_S. The phase transition between them, taking place around 326 K, is associated with sliding at the central inter­faces of the hydro­phobic regions in the crystal, leaving the hydrogen-bonding pattern unperturbed. For the high-temperature α phase, only a structure of rather low quality has been available [*R* factor = 0.118, no H-atom coordinates; Taniguchi *et al.* (1980 ▶). *Bull. Chem. Soc. Jpn*, **53**, 803–804]. We here present accurate structural data for this polymorph [*R*(F) = 0.049], which are compared with other related amino acid structures with similar properties. We report for the first time that the side chain of this phase has a minor disorder component [occupancy 0.0491 (18)] with a *gauche*+ rather than a *gauche*− conformation for the N—C—C—C group. In the crystal of the title compound, N—H⋯O hydrogen bonds link the mol­ecules into (100) sheets.

## Abstract 2

An accurate redetermination of α-dl-me­thio­nine provides coordinates for the H atoms, detailed hydrogen-bond geometries and reveals that the side chain is disordered over a major (95%) and a minor (5%) position.

## Chemical context   

The racemates of amino acids with linear side chains display a series of unique phase transitions that involve sliding of neighboring mol­ecular bilayers compared to each other. Such behavior has been observed for dl-amino­butyric acid (dl-Abu, *R* = –CH_2_CH_3_; Görbitz *et al.*, 2012 ▶), dl-norvaline (dl-Nva, –CH_2_CH_2_CH_3_; Görbitz, 2011 ▶), dl-norleucine (dl-Nle, –CH_2_CH_2_CH_2_CH_3_; Coles *et al.*, 2009 ▶) and dl-me­thio­nine (dl-Met, –CH_2_CH_2_SCH_3_). Two phase transitions have been found for each of the three nonstandard amino acids. For dl-Met, only a single transition is known < 400 K, occurring at approximately 326 K from the β (low *T*) to the α form (high *T*). Both phases were originally described by Mathieson (1952 ▶), with *R* factors > 0.20, and were subject to redeter­min­ations by Taniguchi *et al.* (1980 ▶) at room temperature (*R* = 0.088) and 333 K (*R* = 0.118). The β form was subsequently redetermined at 105 K (*R* = 0.041; Alagar *et al.*, 2005 ▶; refcode DLMETA05 in the Cambridge Structual Database, Version 5.35; Allen, 2002 ▶). α-dl-Met, (I), however, remained one of the few structures of the standard amino acids for which no high-precision experimental data were available (Görbitz, 2015 ▶). We here provide a detailed description of this polymorph, obtained from a single-crystal X-ray diffraction investigation at 340 K.
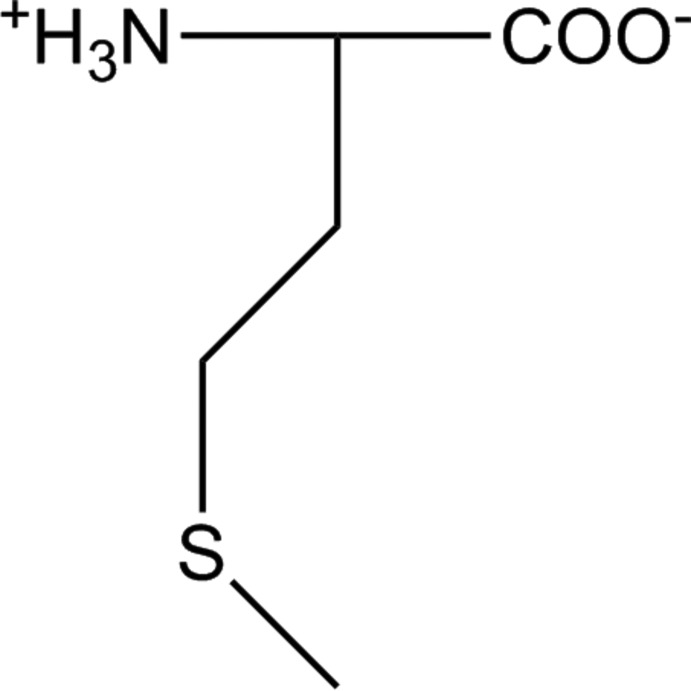


## Structural commentary   

**Figure 1:**
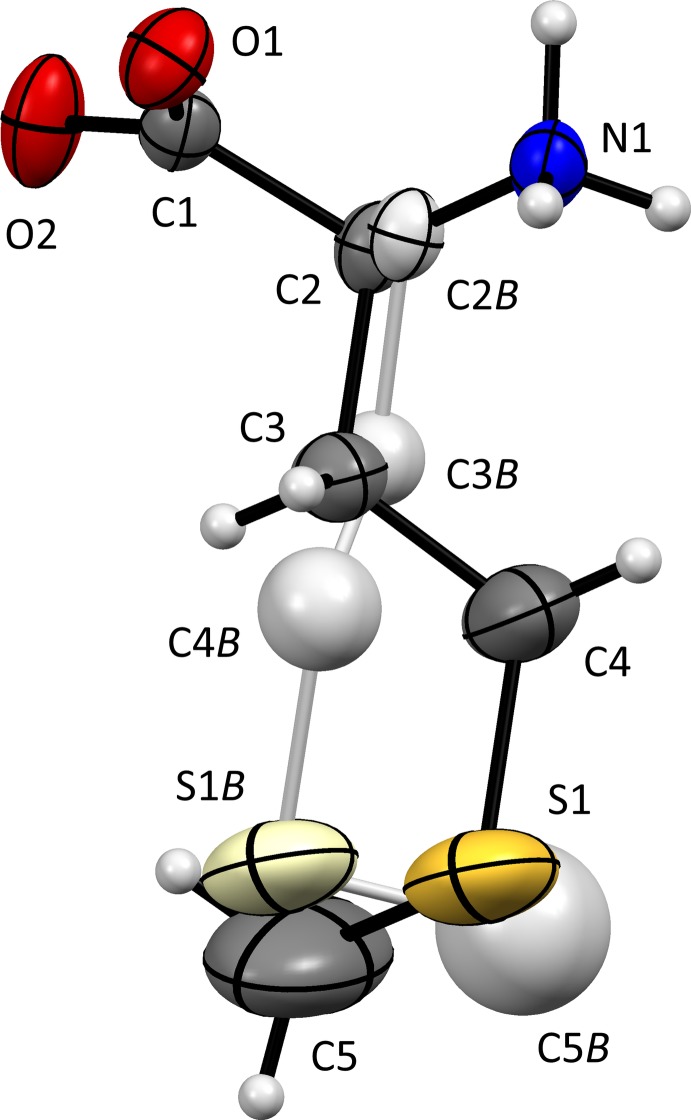
The mol­ecular structure of (I), with 50% probability displacement ellipsoids and atomic numbering indicated. The l-enanti­omer was used as the asymmetric unit, d-enanti­omers being generated by symmetry. The minor side-chain orientation [occupancy 0.0491 (18)], with N1—C2*B*—C3*B*—C4*B* in a *gauche*+ rather than a *gauche*− orientation (Table 1 ▶), is shown in a lighter colour.

The mol­ecular structure of (I) is shown in Fig. 1 ▶. Despite the above-room-temperature conditions, thermal vibrations are comparatively modest. A previously undetected minor conformation with χ^1^(N1—C2*B*—C3*B*—C4*B*) in a *gauche*+ orientation (Table 1 ▶) has occupancy 0.0491 (18). If the presence of this rotamer is neglected, the refinement converges at *R* = 0.0586 rather than 0.0490. Disorder is extensive for all known phases of dl-Abu and dl-Nva, so it is not unexpected that it is observed here for dl-Met.

**Figure 2:**
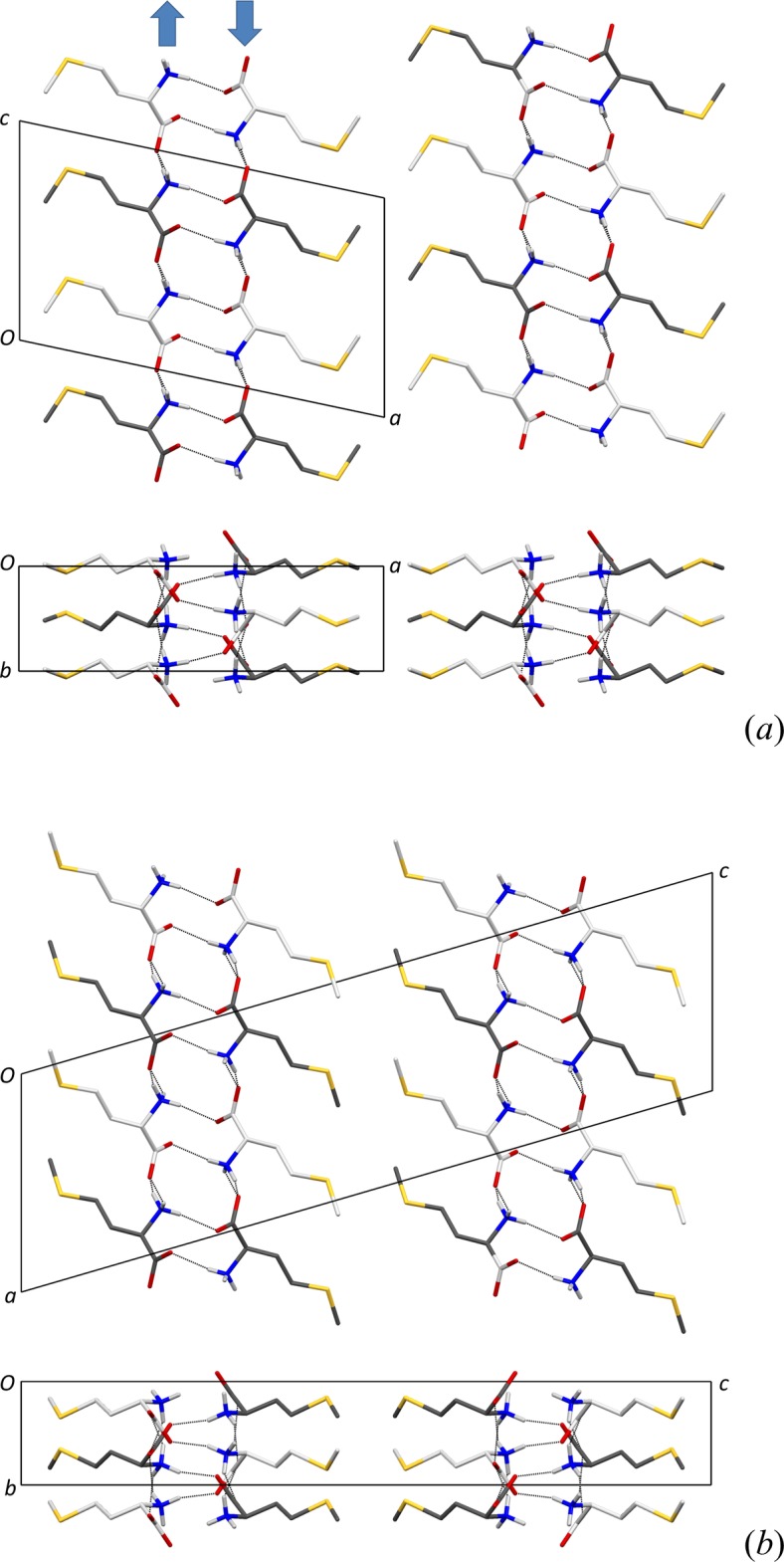
(*a*) The crystal packing of (I), viewed along the monoclinic *b* axis (top) and the *c* axis (bottom). The minor side-chain conformation is not shown, and H atoms bonded to C have been omitted for clarity. l-Met and d-Met mol­ecules are shown with light- and dark-grey C atoms, respectively. The blue arrows show the directions of C2—N bond vectors within each of the two sheets constituting a hydrogen-bonded layer. (*b*) Corresponding views for β-dl-Met at 105 K (Alagar *et al.*, 2005 ▶).

The crystal packing of (I) is shown in Fig. 2 ▶(*a*) and may be compared with the structure of β-dl-Met in Fig. 2 ▶(*b*) (Alagar *et al.*, 2005 ▶). The difference between the two forms is not limited to the obvious conformational change for the C3—C4—S—C5 torsion angle, which is *trans* for the β form, but involves a large shift along the 9.8 Å axis and also the characteristic translation half a unit-cell length along the 4.7 Å axis. Notably, hydrogen bonding is virtually unaffected by these displacements. Compared to the 105 K data, N1⋯O2 distances in Table 1 ▶ are 0.03 Å longer, while N1⋯O1 is 0.01 Å shorter. All H⋯*A* distances surprisingly appear to get shorter at 340 K, but this is an artefact resulting from different ways of handling the amino group (Görbitz, 2014 ▶). In the refinement of β-dl-Met, this group was fixed with idealized geometry and a perfectly staggered orientation, while we find, upon relaxing the positional parameteres for all three H atoms, a 14° counterclockwise rotation (for the l-enanti­omer) that serves to give three shorter and more linear inter­actions.

## Supra­molecular features   

**Figure 3:**
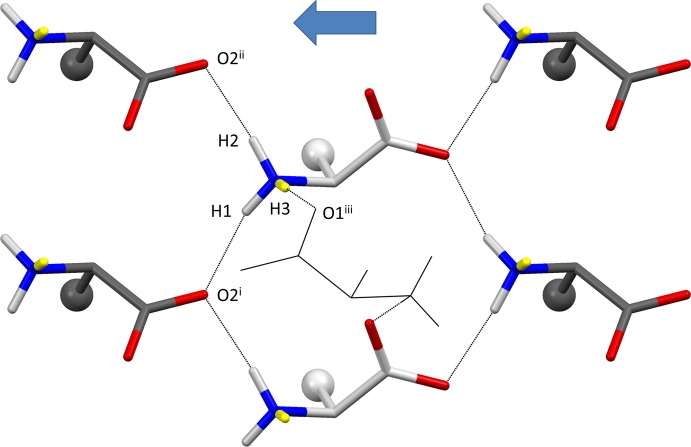
Hydrogen-bonded sheet of (I). Colour coding as in Fig. 2 ▶, except that H3 atoms connecting sheets appear in yellow. The side chains are shown as small spheres. A single l-Met mol­ecule of the adjacent sheet is shown in black wireframe representation. O2^i^ is at (*x*, −*y* + 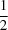, *z* − 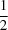), O2^ii^ at (*x*, −*y* + 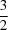, *z* − 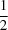) and O1^iii^ at (−*x* + 1, *y* − 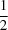, −*z* + 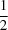) (Table 2 ▶). The blue arrow has the same meaning as in Fig. 2 ▶.

Hydrogen-bond geometries are listed in Table 2 ▶. The hydrogen-bonding patterns of all compounds discussed here belong to the ld–ld type (Görbitz *et al.*, 2009 ▶), normally observed for racemates and quasiracemates where at least one of the side chains (for the l- or the d-enanti­omer) is linear and leucine, with an isobutyl side chain, is not involved (Görbitz *et al.*, 2009 ▶). Apart from a weak C^α^—H⋯O contact along the *b* axis, all inter­molecular inter­actions within a single sheet involve amino acids of opposite chirality (Fig. 3 ▶); two N—H⋯O inter­actions between amino acids of the same chirality serve to link the adjacent anti­parallel sheets that form a double-sheet hydrogen-bonded layer.

## Synthesis and crystallization   

From a saturated solution of dl-Met in water (approximately 30 mg ml^−1^) 50 µl was pipetted into a 40 × 8 mm test tube, which was then sealed with parafilm. A small hole was pricked in the parafilm and the tube placed inside a larger test tube filled with 2 ml of aceto­nitrile. The system was ultimately capped and left for 5 d at 293 K. Suitable single crystals in the shape of plates formed as the organic solvent diffused into the aqueous solution.

## Refinement   

Crystal data, data collection and structure refinement details are summarized in Table 3 ▶. *U*_iso_ values for C*nB* atoms (*n* = 3–5) belonging to the minor side-chain conformation with occupancy 0.0491 (18) were fixed at the *U*_eq_ values of the corresponding C*n* atom of the major conformation, while S1*B* was constrained to have the same set of anisotropic displace­ment parameters as S1. A similar procedure was undertaken for C2*B* and C2. Coordinates were refined for amino H atoms; other H atoms were positioned with idealized geometry with fixed C—H = 0.96 (meth­yl), 0.97 (methyl­ene) or 0.98 Å (methine). *U*_iso_(H) values were set at 1.2*U*_eq_ of the carrier atom or at 1.5*U*_eq_ for methyl and amino groups.

## Supplementary Material

Crystal structure: contains datablock(s) I, global. DOI: 10.1107/S1600536814022211/hb7288sup1.cif

Structure factors: contains datablock(s) I. DOI: 10.1107/S1600536814022211/hb7288Isup2.hkl

Supporting information file. DOI: 10.1107/S1600536814022211/hb7288Isup3.cml

CCDC reference: 1028063

Additional supporting information:  crystallographic information; 3D view; checkCIF report

## Figures and Tables

**Table 1:** Selected torsion angles ()

N1C2C3C4	59.3(4)	N1C2*B*C3*B*C4*B*	73(8)
C2C3C4S1	176.7(2)	C2*B*C3*B*C4*B*S1*B*	178(5)
C3C4S1C5	69.4(3)	C3*B*C4*B*S1*B*C5*B*	60(3)

**Table 2:** Hydrogen-bond geometry (, )

*D*H*A*	*D*H	H*A*	*D* *A*	*D*H*A*
N1H1O2^i^	0.88(3)	1.95(3)	2.812(2)	164(2)
N1H2O2^ii^	0.92(3)	1.94(3)	2.843(2)	168(2)
N1H3O1^iii^	0.93(3)	1.86(3)	2.785(2)	171(2)
C2H21O1^iv^	0.98	2.46	3.264(3)	140

Symmetry codes: (i) 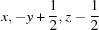; (ii) 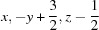; (iii) 
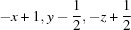
; (iv) 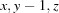.

**Table 3:** Experimental details

Crystal data	
Chemical formula	C_5_H_11_NO_2_S
*M*_r_	149.21
Crystal system, space group	Monoclinic, *P*2_1_/*c*
Temperature (K)	340
*a*, *b*, *c* ()	16.811(5), 4.7281(14), 9.886(3)
()	101.950(7)
*V* (^3^)	768.7(4)
*Z*	4
Radiation type	Mo *K*
(mm^1^)	0.35
Crystal size (mm)	0.62 0.55 0.13

Data collection	
Diffractometer	Bruker D8 Vantage single crystal CCD
Absorption correction	Multi-scan (*SADABS*; Bruker, 2013 ▶)
*T*_min_, *T*_max_	0.819, 1.000
No. of measured, independent and observed [*I* > 2(*I*)] reflections	15046, 1513, 1332
*R*_int_	0.041
(sin /)_max_ (^1^)	0.617

Refinement	
*R*[*F* ^2^ > 2(*F* ^2^)], *wR*(*F* ^2^), *S*	0.049, 0.129, 1.07
No. of reflections	1513
No. of parameters	107
No. of restraints	9
H-atom treatment	H atoms treated by a mixture of independent and constrained refinement
_max_, _min_ (e ^3^)	0.27, 0.29

Computer programs: *APEX2* and *SAINT* (Bruker, 2013 ▶), *SHELXS2013* and *SHELXL2013* (Sheldrick, 2008 ▶) and *Mercury* (Macrae *et al.*, 2008 ▶).

## supplementary crystallographic information

### Crystal data

**Table:**

C_5_H_11_NO_2_S	*F*(000) = 320
*M**_r_* = 149.21	*D*_x_ = 1.289 Mg m^−^^3^
Monoclinic, *P*2_1_/*c*	Mo *K*α radiation, λ = 0.71073 Å
*a* = 16.811 (5) Å	Cell parameters from 9952 reflections
*b* = 4.7281 (14) Å	θ = 2.5–28.3°
*c* = 9.886 (3) Å	µ = 0.35 mm^−^^1^
β = 101.950 (7)°	*T* = 340 K
*V* = 768.7 (4) Å^3^	Plate, colourless
*Z* = 4	0.62 × 0.55 × 0.13 mm

### Data collection

**Table:**

Bruker D8 Vantage single crystal CCD diffractometer	1513 independent reflections
Radiation source: fine-focus sealed tube	1332 reflections with *I* > 2σ(*I*)
Graphite monochromator	*R*_int_ = 0.041
Detector resolution: 8.3 pixels mm^-1^	θ_max_ = 26.0°, θ_min_ = 2.5°
Sets of exposures each taken over 0.5° ω rotation scans	*h* = −20→20
Absorption correction: multi-scan (*SADABS*; Bruker, 2013)	*k* = −5→5
*T*_min_ = 0.819, *T*_max_ = 1.000	*l* = −12→12
15046 measured reflections	

### Refinement

**Table:**

Refinement on *F*^2^	9 restraints
Least-squares matrix: full	Hydrogen site location: inferred from neighbouring sites
*R*[*F*^2^ > 2σ(*F*^2^)] = 0.049	H atoms treated by a mixture of independent and constrained refinement
*wR*(*F*^2^) = 0.129	*w* = 1/[σ^2^(*F*_o_^2^) + (0.0499*P*)^2^ + 0.5391*P*] where *P* = (*F*_o_^2^ + 2*F*_c_^2^)/3
*S* = 1.07	(Δ/σ)_max_ < 0.001
1513 reflections	Δρ_max_ = 0.27 e Å^−^^3^
107 parameters	Δρ_min_ = −0.29 e Å^−^^3^

### Special details

**Table:**

Geometry. All e.s.d.'s (except the e.s.d. in the dihedral angle between two l.s. planes) are estimated using the full covariance matrix. The cell e.s.d.'s are taken into account individually in the estimation of e.s.d.'s in distances, angles and torsion angles; correlations between e.s.d.'s in cell parameters are only used when they are defined by crystal symmetry. An approximate (isotropic) treatment of cell e.s.d.'s is used for estimating e.s.d.'s involving l.s. planes.
Refinement. Disorder, two side chain orientations.

### Fractional atomic coordinates and isotropic or equivalent isotropic displacement parameters (Å^2^)

**Table:**

	*x*	*y*	*z*	*U*_iso_*/*U*_eq_	Occ. (<1)
N1	0.59254 (10)	0.4431 (4)	0.14177 (16)	0.0345 (4)	
H1	0.6057 (14)	0.310 (5)	0.088 (2)	0.052*	
H2	0.5958 (14)	0.614 (6)	0.099 (2)	0.052*	
H3	0.5381 (16)	0.412 (5)	0.144 (2)	0.052*	
O1	0.56499 (8)	0.8214 (3)	0.32823 (13)	0.0414 (4)	
O2	0.62423 (10)	0.5530 (3)	0.50516 (13)	0.0492 (4)	
C1	0.60684 (11)	0.6163 (4)	0.37937 (17)	0.0305 (4)	
C2	0.64538 (12)	0.4338 (8)	0.2836 (3)	0.0307 (7)	0.9509 (18)
H21	0.6505	0.2385	0.3175	0.037*	0.9509 (18)
C3	0.73009 (12)	0.5543 (5)	0.2799 (2)	0.0423 (5)	0.9509 (18)
H31	0.7240	0.7509	0.2515	0.051*	0.9509 (18)
H32	0.7631	0.5497	0.3728	0.051*	0.9509 (18)
C4	0.77480 (16)	0.4004 (7)	0.1849 (3)	0.0697 (8)	0.9509 (18)
H41	0.7407	0.3955	0.0929	0.084*	0.9509 (18)
H42	0.7840	0.2067	0.2165	0.084*	0.9509 (18)
S1	0.87076 (5)	0.5570 (3)	0.17503 (10)	0.0907 (4)	0.9509 (18)
C5	0.9285 (2)	0.4833 (15)	0.3418 (5)	0.140 (2)	0.9509 (18)
H51	0.9820	0.5616	0.3507	0.211*	0.9509 (18)
H52	0.9324	0.2823	0.3552	0.211*	0.9509 (18)
H53	0.9025	0.5659	0.4100	0.211*	0.9509 (18)
C2B	0.6365 (12)	0.435 (16)	0.258 (10)	0.0307 (7)	0.0491 (18)
H22B	0.6223	0.2457	0.2854	0.037*	0.0491 (18)
C3B	0.7299 (13)	0.406 (8)	0.293 (4)	0.042*	0.0491 (18)
H33B	0.7467	0.3580	0.3903	0.050*	0.0491 (18)
H34B	0.7450	0.2491	0.2403	0.050*	0.0491 (18)
C4B	0.7757 (10)	0.665 (6)	0.265 (5)	0.069*	0.0491 (18)
H43B	0.7592	0.8227	0.3153	0.083*	0.0491 (18)
H44B	0.7602	0.7089	0.1669	0.083*	0.0491 (18)
S1B	0.8843 (9)	0.632 (5)	0.311 (2)	0.0907 (4)	0.0491 (18)
C5B	0.902 (2)	0.347 (12)	0.205 (7)	0.138*	0.0491 (18)
H54B	0.9590	0.3181	0.2150	0.207*	0.0491 (18)
H55B	0.8781	0.3901	0.1100	0.207*	0.0491 (18)
H56B	0.8770	0.1791	0.2319	0.207*	0.0491 (18)

### Atomic displacement parameters (Å^2^)

**Table:**

	*U*^11^	*U*^22^	*U*^33^	*U*^12^	*U*^13^	*U*^23^
N1	0.0413 (9)	0.0351 (9)	0.0287 (8)	−0.0045 (7)	0.0110 (7)	−0.0053 (7)
O1	0.0466 (8)	0.0371 (8)	0.0425 (8)	0.0092 (6)	0.0137 (6)	−0.0001 (6)
O2	0.0812 (11)	0.0411 (8)	0.0276 (7)	0.0022 (7)	0.0165 (7)	0.0011 (6)
C1	0.0359 (9)	0.0274 (8)	0.0306 (9)	−0.0060 (7)	0.0125 (7)	−0.0012 (7)
C2	0.0386 (10)	0.0273 (9)	0.0269 (19)	0.0018 (9)	0.0084 (9)	0.0009 (10)
C3	0.0366 (11)	0.0439 (12)	0.0478 (12)	−0.0009 (9)	0.0122 (9)	−0.0041 (10)
C4	0.0514 (14)	0.080 (2)	0.0857 (19)	−0.0088 (14)	0.0323 (14)	−0.0238 (16)
S1	0.0536 (5)	0.1247 (9)	0.1036 (7)	−0.0132 (5)	0.0393 (4)	−0.0009 (6)
C5	0.057 (2)	0.234 (6)	0.127 (4)	0.021 (3)	0.013 (2)	0.005 (4)
C2B	0.0386 (10)	0.0273 (9)	0.0269 (19)	0.0018 (9)	0.0084 (9)	0.0009 (10)
S1B	0.0536 (5)	0.1247 (9)	0.1036 (7)	−0.0132 (5)	0.0393 (4)	−0.0009 (6)

### Geometric parameters (Å, º)

**Table:**

N1—C2B	1.23 (8)	S1—C5	1.766 (5)
N1—C2	1.497 (3)	C5—H51	0.9600
N1—H1	0.88 (3)	C5—H52	0.9600
N1—H2	0.92 (3)	C5—H53	0.9600
N1—H3	0.93 (3)	C2B—C3B	1.542 (6)
O1—C1	1.242 (2)	C2B—H22B	0.9800
O2—C1	1.253 (2)	C3B—C4B	1.505 (6)
C1—C2	1.521 (4)	C3B—H33B	0.9700
C1—C2B	1.63 (10)	C3B—H34B	0.9700
C2—C3	1.541 (3)	C4B—S1B	1.794 (6)
C2—H21	0.9800	C4B—H43B	0.9700
C3—C4	1.506 (3)	C4B—H44B	0.9700
C3—H31	0.9700	S1B—C5B	1.765 (7)
C3—H32	0.9700	C5B—H54B	0.9600
C4—S1	1.796 (3)	C5B—H55B	0.9600
C4—H41	0.9700	C5B—H56B	0.9600
C4—H42	0.9700		
			
C2B—N1—H1	112 (4)	C5—S1—C4	101.2 (2)
C2—N1—H1	111.8 (15)	S1—C5—H51	109.5
C2B—N1—H2	112 (3)	S1—C5—H52	109.5
C2—N1—H2	112.0 (14)	H51—C5—H52	109.5
H1—N1—H2	108 (2)	S1—C5—H53	109.5
C2B—N1—H3	112 (3)	H51—C5—H53	109.5
C2—N1—H3	111.9 (14)	H52—C5—H53	109.5
H1—N1—H3	106 (2)	N1—C2B—C3B	127 (6)
H2—N1—H3	107 (2)	N1—C2B—C1	117 (4)
O1—C1—O2	125.88 (17)	C3B—C2B—C1	110 (5)
O1—C1—C2	117.89 (17)	N1—C2B—H22B	98.6
O2—C1—C2	116.11 (18)	C3B—C2B—H22B	98.6
O1—C1—C2B	110 (2)	C1—C2B—H22B	98.6
O2—C1—C2B	124 (2)	C4B—C3B—C2B	114.8 (7)
N1—C2—C1	108.6 (2)	C4B—C3B—H33B	108.6
N1—C2—C3	109.8 (2)	C2B—C3B—H33B	108.6
C1—C2—C3	108.7 (2)	C4B—C3B—H34B	108.6
N1—C2—H21	109.9	C2B—C3B—H34B	108.6
C1—C2—H21	109.9	H33B—C3B—H34B	107.5
C3—C2—H21	109.9	C3B—C4B—S1B	114.5 (6)
C4—C3—C2	114.8 (3)	C3B—C4B—H43B	108.6
C4—C3—H31	108.6	S1B—C4B—H43B	108.6
C2—C3—H31	108.6	C3B—C4B—H44B	108.6
C4—C3—H32	108.6	S1B—C4B—H44B	108.6
C2—C3—H32	108.6	H43B—C4B—H44B	107.6
H31—C3—H32	107.5	C5B—S1B—C4B	101.5 (5)
C3—C4—S1	113.9 (2)	S1B—C5B—H54B	109.5
C3—C4—H41	108.8	S1B—C5B—H55B	109.5
S1—C4—H41	108.8	H54B—C5B—H55B	109.5
C3—C4—H42	108.8	S1B—C5B—H56B	109.5
S1—C4—H42	108.8	H54B—C5B—H56B	109.5
H41—C4—H42	107.7	H55B—C5B—H56B	109.5
			
N1—C2—C3—C4	−59.3 (4)	O1—C1—C2—N1	−29.4 (3)
C2—C3—C4—S1	176.7 (2)	O2—C1—C2—N1	154.35 (18)
C3—C4—S1—C5	69.4 (3)	O1—C1—C2—C3	90.0 (2)
N1—C2B—C3B—C4B	73 (8)	O2—C1—C2—C3	−86.2 (2)
C2B—C3B—C4B—S1B	178 (5)	C1—C2—C3—C4	−178.0 (2)
C3B—C4B—S1B—C5B	60 (3)	C1—C2B—C3B—C4B	−78 (5)

### Hydrogen-bond geometry (Å, º)

**Table:**

*D*—H···*A*	*D*—H	H···*A*	*D*···*A*	*D*—H···*A*
N1—H1···O2^i^	0.88 (3)	1.95 (3)	2.812 (2)	164 (2)
N1—H2···O2^ii^	0.92 (3)	1.94 (3)	2.843 (2)	168 (2)
N1—H3···O1^iii^	0.93 (3)	1.86 (3)	2.785 (2)	171 (2)
C2—H21···O1^iv^	0.98	2.46	3.264 (3)	140

Symmetry codes: (i) *x*, −*y*+1/2, *z*−1/2; (ii) *x*, −*y*+3/2, *z*−1/2; (iii) −*x*+1, *y*−1/2, −*z*+1/2; (iv) *x*, *y*−1, *z*.
